# Carbon Fiber-Based Vitrimer Composites: A Path toward Current Research That Is High-Performing, Useful, and Sustainable

**DOI:** 10.3390/ma17133265

**Published:** 2024-07-02

**Authors:** Vishal Kumar, Wenbin Kuang, Leonard S. Fifield

**Affiliations:** Pacific Northwest National Laboratory, Richland, WA 99354, USA; vishal.kumar@pnnl.gov (V.K.); wenbin.kuang@pnnl.gov (W.K.)

**Keywords:** processable thermoset, circular material, dynamic covalent bonds, recyclable polymer

## Abstract

In the polymeric material industry, thermosets and related composites have played a substantial role in the production of rubber and plastics. One important subset of these is thermoset composites with carbon reinforcement. The incorporation of carbon fillers and fibers gives polymeric materials improved electrical and mechanical properties, among other benefits. However, the covalently crosslinked network of thermosets presents significant challenges for recycling and reprocessing because of its intractable nature. The introduction of vitrimer materials opens a new avenue to produce biodegradable and recyclable thermosets. Carbon-reinforced vitrimer composites are pursued for high-performance, long-lasting materials with attractive physical properties, the ability to be recycled and processed, and other features that respond uniquely to stimuli. The development of carbon-reinforced vitrimer composites over the last few years is summarized in this article. First, an overview of vitrimers and the methods used to prepare carbon fiber-reinforced vitrimer composites is provided. Because of the vitrimer nature of such composites, reprocessing, healing, and recycling are viable ways to greatly extend their service life; these approaches are thoroughly explained and summarized. The conclusion is our prediction for developing carbon-based vitrimer composites.

## 1. Introduction

Thermoset polymers and their composites such as epoxies, vinyl ester, polyimides, and polyurethanes, are widely used in practically every aspect of our daily life as well as in high-tech businesses, including everything from electronics and cars to shoes and furnishings. Owing to their permanently crosslinked network, thermosets have unique advantages over thermoplastics, such as exceptional dimensional stability, high thermal resistance, chemical resistance, and excellent mechanical characteristics, which make them desirable in many structural applications [1]. To further enhance mechanical properties, promote thermal and electrical conductivity, and reduce weight, thermoset composites are frequently produced using carbon-based fillers such as carbon fibers, carbon nanotubes (CNTs), and graphene [2,3,4,5]. Carbon-reinforced materials are widely employed in a growing number of important sectors. Particularly, carbon fibers have been widely used in many industries to produce lightweight materials and increase mechanical strength [6]; carbon black has been used to reduce thermal damage due to its good thermal conductivity; and CNTs have been combined with thermoset polymers to develop high strength materials, electronics, etc., due to their outstanding mechanical, electrical, optical, and thermal characteristics [7,8]. A single layer of graphite (graphene), discovered in 2004, has been progressively investigated as a new stage in polymeric composites for different applications such as solar cells, sensors, capacitors, etc. [9,10]. Without a doubt, such carbon materials will continue to play a vital role in thermoset applications and in scientific studies on functional polymers, which will help to grow markets and applications [11,12,13].

Carbon-reinforced thermoset composites face significant challenges in recycling and reprocessing, just as conventional thermosets do. This results in a practically zero recycle rate for these initially high value materials. Unlike thermoplastics, thermosets generally decompose before melting or flowing at elevated temperatures and do not dissolve in solvents. In addition to waste, recycling of fillers and reinforcement is a challenging but critical issue that needs resolution given the high cost and demanding manufacturing processes associated with high-performance carbon-based filler materials. Meanwhile, the fact that conventional thermoset composites are difficult to repair if cracks or fractures arise during use is another significant drawback.

Much work has been performed over the last 2 years to find novel materials that have both the longevity and strength of thermoset polymers with the reprocessing and recycling abilities of traditional thermoplastics [14,15]. The most interesting and promising concept on this front has been the identification of dynamically crosslinked systems that can change shape when heated while maintaining crosslink density. This idea was first put forward by Leibler and colleagues in 2011 [16] and has quickly led to extensive study of the “third kind of polymeric material”, vitrimers, and their carbon-reinforced composites [17,18,19,20,21].

The wide range of uses and ongoing research into carbon-reinforced thermosets have led to the progress of new carbon-reinforced vitrimer composites that are reusable, useful, and have high performance. Specifically, the advancement of carbon-reinforced vitrimer composites make it possible to recycle and reuse the composite itself. It also facilitates recycling of the carbon reinforcement, enabling a possible beneficial method to deal with waste produced from thermoset composites [22,23]. As already stated, many carbon materials have advantageous electrical and thermal optical qualities. These can be added to vitrimers to increase their value as strong and adaptable materials. The developed materials can be deployed as shape memory polymers, actuators, sensors, and other applications in new and creative ways, which is why they are getting so much attention.

This review will emphasize current advancements in carbon fiber-based vitrimer composites. This is an area that is growing in prominence but has received limited attention until now. To provide an explicit understanding of vitrimers and vitrimer composites, a concise introduction to these substances will be initially provided. Further elaboration on the principles and mechanisms of vitrimers can be found in the published evaluations [14,17,24,25,26,27]. In the third segment, a summary of the categorization and fabrication methodologies for carbon fiber-based vitrimer composites is provided. After that, a thorough discussion will be presented of the use of carbon fiber-based vitrimer composites as recyclable and long-lasting materials, a central focus of their development. Simultaneously, the incorporation of carbon fiber into vitrimers imparts an array of enticing new functionalities that will be succinctly reviewed and summarized in the following section. In the concluding section, a perspective and conclusion are provided concerning the present unresolved matters and forthcoming developments for vitrimers and vitrimer composites.

## 2. Vitrimers—General Introduction

Vitrimers are a captivating category of materials that challenge the distinction between thermoplastics and conventional thermosets. Vitrimers are dynamic polymer networks that may rearrange their chemical bonds and molecular structure in response to certain stimuli, combining the characteristics of “vitreous” (glass-like) and rigid materials. This feature distinguishes them from traditional thermosetting polymers that experience permanent crosslinking during the curing process. What makes vitrimers different is their dynamic exchange of covalent bonds, allowing them to undergo reshaping, repair, and recycling processes akin to those of thermoplastic materials. This changeable quality comes from reversible chemical bonding in the polymer network, like transesterification or exchange reactions. These reactions allow the material to flow and rearrange itself in certain conditions while maintaining its structural integrity. The characteristics arise from the topology of the polymer network changing due to molecular rearrangements, while maintaining the total number of bonds in the network [14].

To provide a more complete explanation of the concept and attributes of vitrimers, it is necessary to introduce an additional term: covalent adaptable network (CAN) [17]. CANs are, in essence, polymer networks composed of elements that form dynamic covalent bonds. Constant breaking and re-forming of dynamic covalent bonds in CANs (typically at high temperatures) permits reorganizing of the polymer network. A wide variety of dynamic covalent reactions, including disulfide exchange [28], transesterification [29], transamination [30,31], and others, have been utilized to produce CANs.

**Figure 1:**
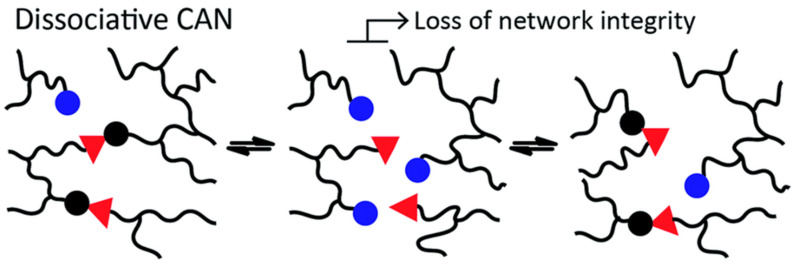
Dissociate bond exchange mechanism. Red triangles and circles (both blue and black) represent reaction sites on polymer chains (black lines) that reversibly bond and de-bond resulting in a dynamic polymer crosslink density. Blue circles represent reaction sites that are available to form new bonds with red triangles and once the new bond is formed, it is marked as a black circle. CAN = covalent adaptable network. Adapted from Ref. [17] with permission, Copyright 2016, from the Royal Society of Chemistry.

**Figure 2:**
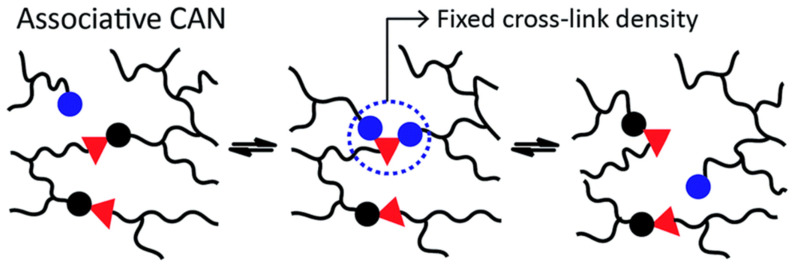
Associative bond exchange mechanism. Red triangles and circles (both blue and black) represent reaction sites on polymer chains (black lines) that reversibly bond and de-bond resulting in a dynamic polymer crosslink density. Blue circles represent reaction sites that are available to initiate a bond exchange reaction on existing bonds between the red triangles and black circles when new bonds are formed resulting a fixed crosslink density. CAN = covalent adaptable network. Adapted from Ref. [17] with permission, Copyright 2016, Royal Society of Chemistry.

Vitrimers exist in an ideal bond exchange state when the breaking of existing bonds and the forming of new ones occurs instantaneously, without passing through an intermediate state. In practice, bond exchange-induced network rearrangement follows a sequential progression that is typically divided into two classes: dissociative and associative exchange as shown in Figure 1 and Figure 2. The dissociative exchange reaction involves sequential breaking and re-forming of dynamic bonds; therefore, the density of crosslinking will diminish significantly during the transitional process, particularly when subjected to high temperatures. Those CANs having Diels–Alder adducts are a prime example; during the retro-Diels–Alder reaction, these adducts dissociate into linear polymers [32]. Therefore, it is commonly assumed that this exchange condition does not meet the connectivity necessities of a vitrimer. However, dissociative CANs can exhibit vitrimer-like characteristics in certain exceptional circumstances (e.g., dissociative networks having greater reaction enthalpy or those requiring catalysts, where the impact of network integrity is insignificant) [14].

Associative networks maintain their network integrity as well as crosslink density throughout the process of bond exchange [33], as shown in Figure 2. The exchange occurs in accordance with the mechanism of bond forming followed by dissociation. The initial vitrimer is produced through deliberate introduction of transesterification, an associative bond exchange, into a network that had been covalently crosslinked. The transesterification catalyst is integrated into bisphenol A and diacid. At higher temperatures (specifically, above the topology freezing transition temperature, Tv), the ester bond exchange can be initiated. The rapid rearrangement of the crosslinked network is accompanied by incremental variation in the viscosity of the polymer network, resembling Arrhenius-like patterns observed in inorganic “glass” [16]. Hence, the substance acquired its nomenclature, “vitrimer”. In the interim, the substance remains intractable. A collection of vitrimers that possess novel functionalities such as reprocessability, recyclability, malleability, self-healing, weldability, transesterification, and disulfide exchange, have been developed following groundbreaking research [25]. Additionally, scholars have been undertaking more comprehensive investigations into the dynamics, principles, and theory underlying vitrimers to perfect their preparation, investigation, and engineering implementations [34,35,36].

**Figure 3:**
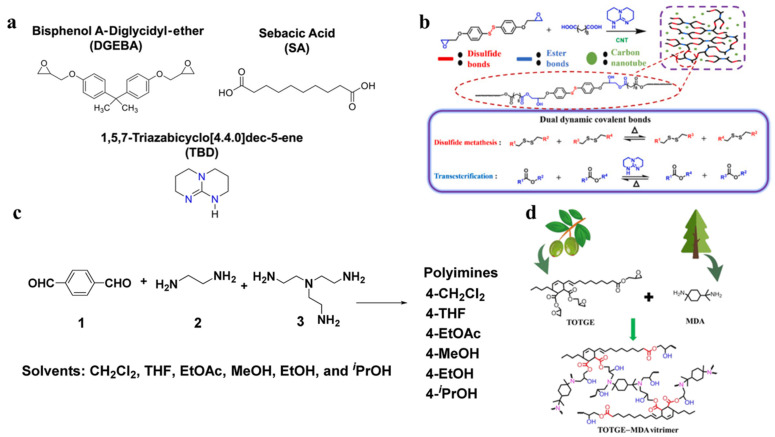
Biobased vitrimer composites (**a**) epoxy vitrimer. Adapted with permission from Ref. [42], Copyright 2023, American Chemical Society. (**b**) Preparation of epoxy vitrimer using dual dynamic covalent bonds, disulfide bonds and ester bonds. Adapted with permission from Ref. [38], Copyright 2021, Elsevier. (**c**) Preparation of malleable thermosets using polyimine. Adapted with permission from Ref. [39], Copyright 2019, Royal Society of Chemistry. (**d**) Synthesis of vitrimer by biomaterials. Adapted from Ref. [51] with permission, Copyright 2021, American Chemical Society.

Epoxy vitrimers are the most frequently used matrices for carbon-based composites, as determined by the principal chemical element [37,38,39,40,41,42]. Polyimine vitrimers are, subsequently, most frequently used vitrimer matrix. Illustrative instances of vitrimer matrix synthesis processes are presented in Figure 3a–d. Additional matrices consist of those composed of rubber-based materials, e.g., ethylene-propylene-diene rubbers [43], styrene-butadiene rubbers [44,45], natural rubbers [46], and polyurethane vitrimers [47]. Bio-based vitrimers, in addition to petroleum-based vitrimers, have been extensively investigated for use in carbon-reinforced composites to meet the increasingly stringent demands of environmentally friendly and sustainable development, such as the examples seen in Figure 3d [48,49,50,51]. Further investigation has been conducted into the potential of vitrimer composites through incorporation of flame-retardant functional groups into the skeleton of the vitrimer network. An illustration of this can be noted in the recent development of carbon fiber/epoxy vitrimer composites that are fire-resistant through the modification of diol derived from phosphaphenanthrene [52,53]. The preparation and design methodologies of vitrimers have been the subject of extensive discussion and evaluation in numerous comprehensive reviews, which offer extensively referenced materials. As a result, the content below will center on innovative and general manufacturing techniques for carbon-filled vitrimer composites.

## 3. Carbon Fiber-Based Vitrimer Composites and Their Preparation Strategies

This article categorizes carbon fiber vitrimer composites, explains production methods, and delivers a summary of the characteristics of the carbon materials that are currently utilized for vitrimer composites to simplify the review and discussion. Because vitrimers can be reprocessed, combining them has opened new options for the thermoset composite fabrication process. We will go over each preparation technique for the carbon-based vitrimer composites.

### 3.1. Carbon Fiber-Based Vitrimer Composites

Carbon fiber is a conventional one-dimensional material known for its high strength-to-weight and stiffness-to-weight performance relative to other fibers such as glass fiber [54,55,56]. It can have a tensile strength of 7 GPa and a modulus of 1000 GPa. Carbon fibers exhibit a low elongation at break lower than 2.5%, strong resistance to creep, and a density ranging from 1.75 to 2.00 g/cm^−3^ [57], making them desirable structural materials for vehicles and airplanes, as well as sports equipment such as motorcycles, bicycles, helmets, and rackets, owing to their high modulus and strength combined with low density. Furthermore, studies on carbon fiber vitrimer composites are consistently emerging in the literature [39,58,59]. 

**Figure 4:**
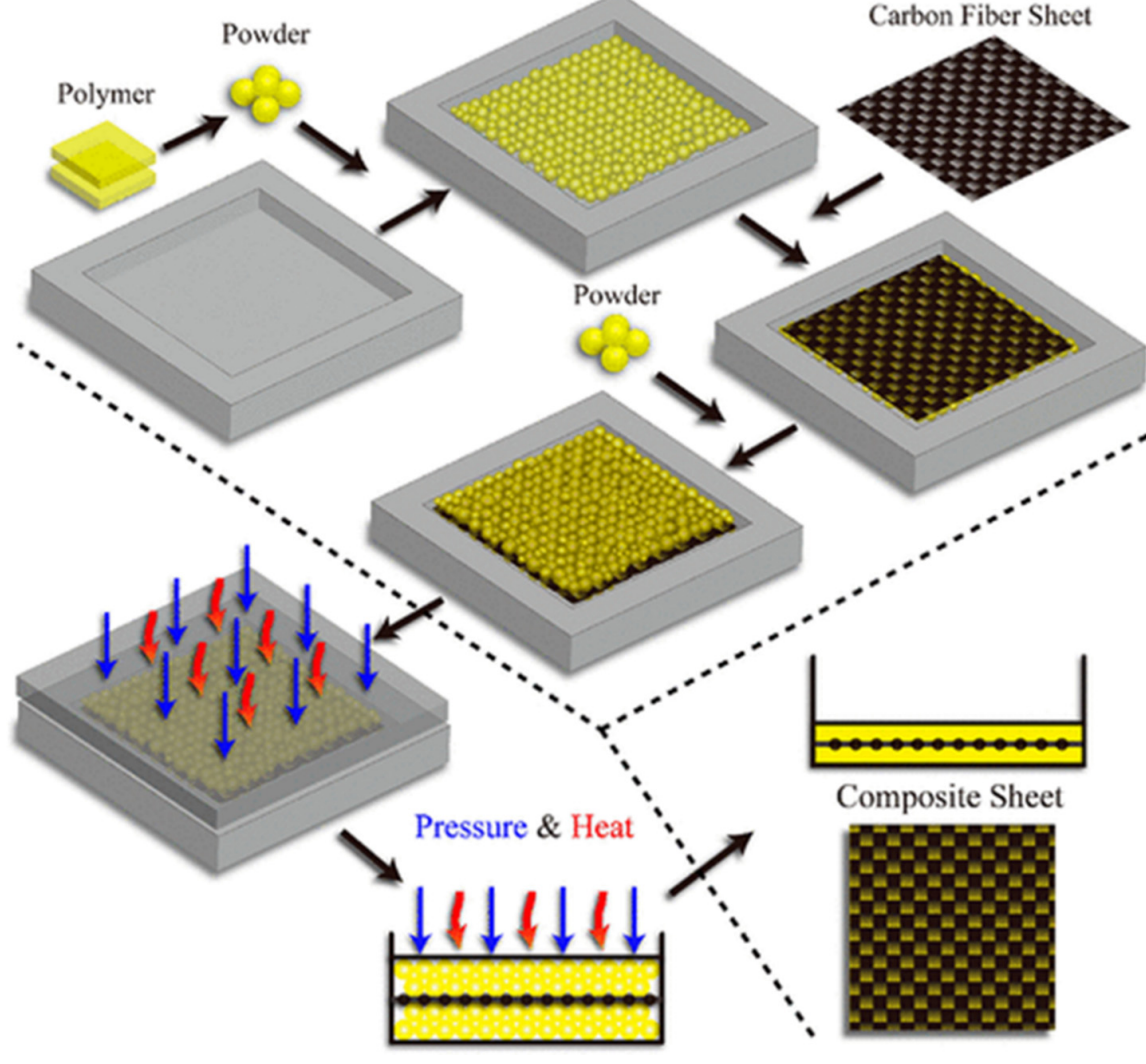
Fabrication process of vitrimer composite from vitrimer powder. Adapted from Ref. [64] with permission, Copyright 2019, American Chemical Society.

Reinforcing carbon fiber is frequently arranged in parallel or interlaced mats, in contrast to other carbon fillers, which are usually in particulate form. It is difficult to reinforce carbon fiber in vitrimers by mixing fibers with precursors [60]. The production of carbon fiber–vitrimer composites is usually accomplished in one of two ways. The first is essentially cast molding of vitrimer solutions on fiber layers or immersing fiber layers in a monomer solution followed by curing [50,61,62,63]. This is like the standard manufacturing process of traditional fiber thermoset composites. Another fabrication technique involves compressing the cured vitrimer (in sheet or powder form) with a fiber layer, utilizing the flexibility of the vitrimer [64,65,66]. Long et al. [64] discovered a rapid technique to produce vitrimer composites by placing carbon fiber in between vitrimer powders and subjecting it to temperatures above Tv. Epoxy vitrimers have been produced using Bisphenol A diglycidyl ether (DGEBA), fatty acid, and zinc acetate (Zn(OAc)_2_) as transesterification catalysts, being a traditional chemical for epoxy vitrimers. The curing of the polymer composites occurs at 130 °C by melting and polymerization. Initially, the polymeric material was pulverized into small granules or powder and applied as a base layer on the mold. A carbon fiber mat was then placed on top of the vitrimer powder, followed by a secondary layer of vitrimer powder on the top of the mat. Ultimately, the material was subjected to hot-pressing at appropriate pressure and temperature. The processing time can be reduced to 1 min at around 1 MPa and around 300 °C, as depicted in Figure 4. 

**Figure 5:**
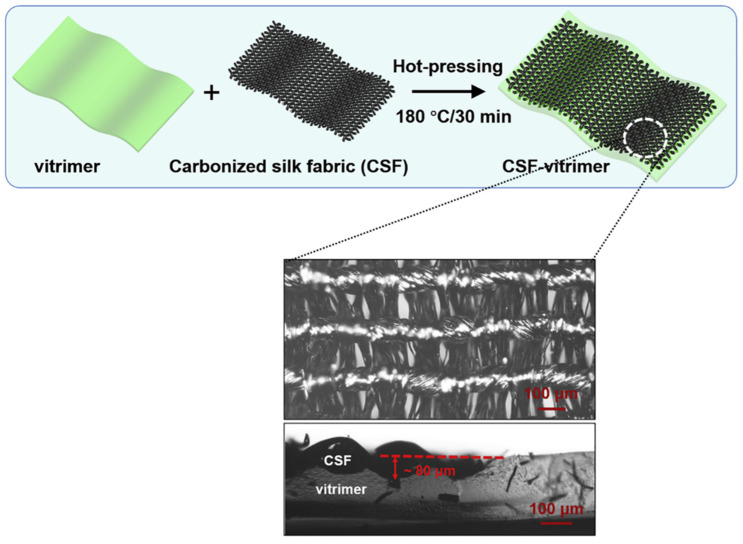
Fabrication of vitrimer composite by hot-pressing and optical microscopic image of a carbonized silk fabric (CSF)-vitrimer composite. Adapted with permission from Ref. [65], Copyright 2021, Elsevier.

The composites obtained after the compression process had a Young’s modulus of around 2.3 GPa and a tensile strength of 41 MPa. In comparison, the pure epoxy vitrimer had a Young’s modulus of approximately 3.8 MPa and a tensile strength of about 2.8 MPa. Molding time, pressure, and temperature can be varied to change the mechanical performance of the product [67]. The carbon fiber can be coupled to the vitrimer directly by hot-pressing it with the fully cured vitrimer. The carbon fiber was intimately integrated into the vitrimer surface as depicted in Figure 5 [65]. 

Carbon fiber composites are frequently used as laminates [68]. Recently, the vitrimer approach enabled the production of cured prepregs that are highly robust during storing and may be bonded to create layered laminates. Manufacturing laminated composites is typically performed by preparing multiple prepreg layers for consolidation in a composite part. These sheets require specific protection and storage conditions. They are stacked and cured under adequate pressure to ensure all the layers are chemically bonded [69]. The covalent network of vitrimers allows their polymeric composites to be chemically fused like metals. Carbon fiber/vitrimer composite laminates are produced by initially creating a single cured layer and subsequently bonding the necessary layers at elevated temperatures above Tv and required pressure [70,71,72]. Laminating carbon-fiber/vitrimer sheets adequately usually requires high temperatures for few hours. Saito et al. [73] recently published a study on a carbon fiber-infused hybrid polyurea/epoxy vitrimer that can be recycled at a relatively low temperature and in a short time. The vitrimer can be reformed at 160 °C and 50 MPa for just 1 min to initiate the rapid exchange reaction of disulfide bonds and the effective polyurea/epoxy chain rearrangement. Reducing processing time may greatly decrease the deterioration and energy usage at elevated temperatures.

### 3.2. Recycling and Reuse of Carbon Fiber Vitrimer Composites: Strategies

**Figure 6:**
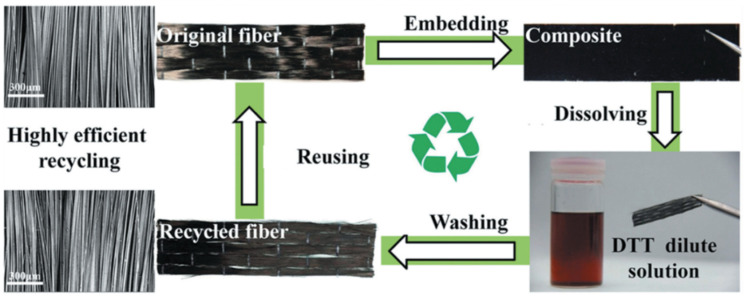
Recycling of carbon fiber-reinforced polymer composites and reusing the product to form new composites. DTT = dithiothreitol. Adapted with permission from Ref. [63], Copyright 2020, Elsevier.

A major goal of fabricating vitrimer-based composites is to enable the recycling of high-performance composite waste, not possible with ordinary thermoset composites. This includes both the polymer matrix and reinforced fillers, which, for standard thermosets, are difficult to reprocess, remold, or reuse due to their permanent crosslinking network structure. Conventional thermoset polymer composite wastes are commonly disposed of by incineration or landfill in a non-sustainable way. In addition, carbon fiber is a costly reinforcement material used in polymer composites. Recycling could provide environmental and economic advantages. Historically, recycling procedures for fillers have involved mechanical grinding of the composites, chemical solvolysis, composite pyrolysis, and other techniques [1,74]. Alternatives for reusing the materials, however, are affected by the high cost, low energy efficiency, and low mechanical characteristics of the regenerated carbon fiber [75]. Exploring vitrimer composites offers a viable solution to these issues. Vitrimer composites can be sustainably utilized through reprocessing via reshaping and remolding, repair of fractured samples, and recycling [63], as depicted in Figure 6. The strategies will be outlined and thoroughly examined below.

### 3.3. Reshaping of Composites

**Figure 7:**
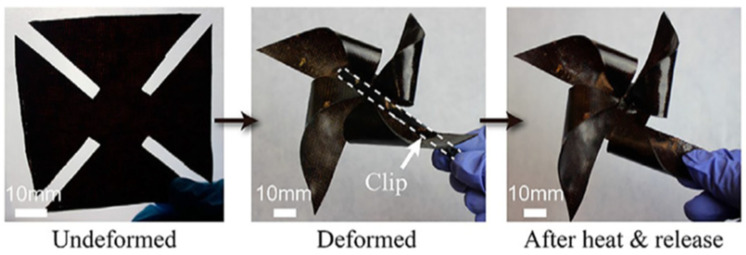
Reshaping a carbon fiber-based vitrimer composite sheet (2D) into a windmill structure (3D). Adapted from Ref. [64] with permission, Copyright 2019, American Chemical Society.

Conventional thermosets cannot be reconfigured after curing, like thermoplastics, unless they are adjusted by cutting or bonding with adhesives. The flexible quality of the vitrimer allows for the simple reprocessing the carbon fiber-based vitrimer composites. However, the shape and structure of a material play a crucial role in determining its function. Therefore, reshaping and remolding can be a simple and effective method to redefine potential applications of the material. Interestingly, vitrimer sheets (planar 2D structure) can be easily reshaped into new complex 3D structures. With appropriate external forces, the vitrimer composites are also able to change into shapes that are challenging to create by traditional molding methods as shown in Figure 7. 

**Figure 8:**
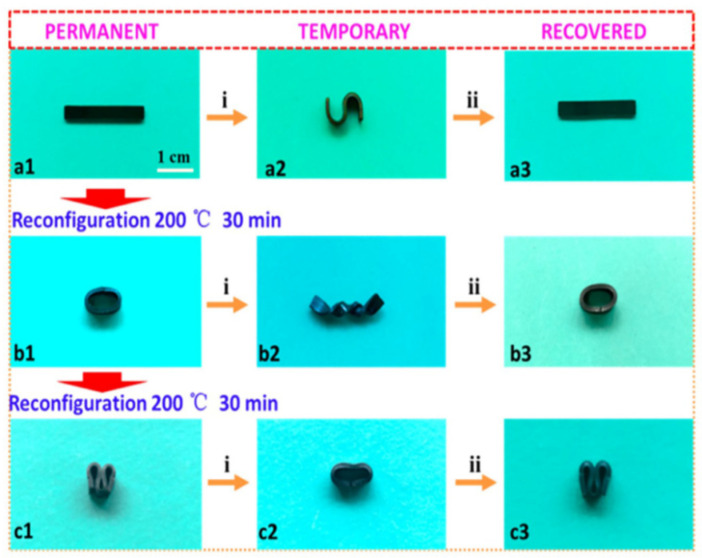
Shape memory effect of a vitrimer graphene composite. The shape fix process above glass transition temperature is represented by “i” and the shape recovery process is represented by “ii”. (**a1**–**a3**) Strip sample qualitative shape change demonstration. (**b1**–**b3**) Reconfiguration of strip into ring, deformation, and recovery. (**c1**–**c3**) Reconfiguration of strip into “M” shape, deformation, and recovery. Adapted from Ref. [76] with permission, Copyright 2016, American Chemical Society.

Utilizing a versatile material for several applications and purposes is highly advantageous for promoting sustainability. By employing direct thermomechanical processing, a single material can attain complex 2D or 3D shapes and undergo form changes through integrating shaping through the shape memory phenomenon in certain vitrimer-based composites. Wang et al. [76] demonstrated samples with memory forms above the glass transition temperature (Tg) and a shape changing process above Tv in a strip composed of vitrimer graphene composites, as depicted in Figure 8.

**Figure 9:**
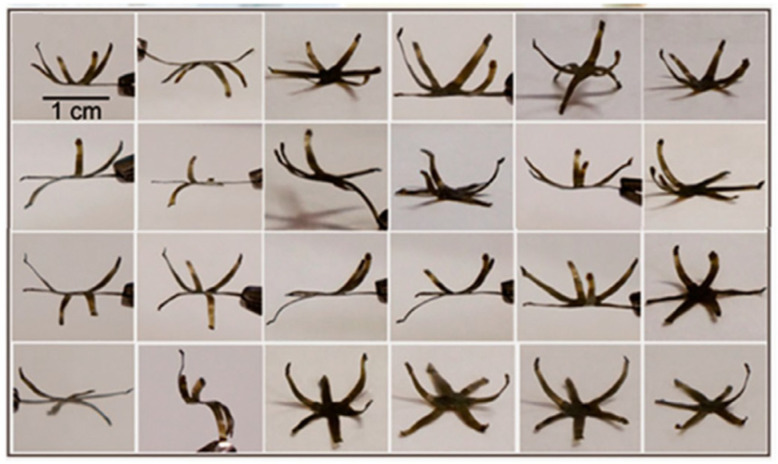
Liquid crystal–vitrimer/carbon nanotube composites reshaped into various 3D structures. Adapted from Ref. [77] with permission, Copyright 2016, American Chemical Society.

Reprocessing liquid crystalline (LC) vitrimer composites tends to create intricate permanent forms and introduce novel actuation modes. The realignment of LC components is influenced by the rearrangement of chains in a polymer matrix due to external forces like compression and extension, resulting in a shift in the actuation mode caused by the LC-isotropic phase transition. In 2016, Yang et al. [77] first presented LC vitrimer composites, specifically epoxy-LC vitrimer CNT composites. The photo-assisted reshaping allowed for a wide range of configurations and intricate shape change patterns, as pictured in Figure 9. 

During the reshaping of carbon fiber-reinforced composites, fiber wrinkling, sliding, and matrix penetration are common phenomena that may occur. The impacts of these phenomena could lead to the creation of empty spaces, creases, protrusions, or an uneven spread of fibers. In an investigation of such effects, Markaide, et al. [78] studied the thermoforming capability of a disulfide-based epoxy vitrimer using three-point bending. Creating flawless thermoforming was challenging due to the elevated viscosities of the vitrimer material. Various types of issues can arise during both bending and profile reshaping processes. Those authors recommended utilizing a vitrimer with lower viscosity at an appropriate temperature for the thermoforming of carbon fiber vitrimer composites.

### 3.4. Repairing Vitrimer Based Composites

**Figure 10:**
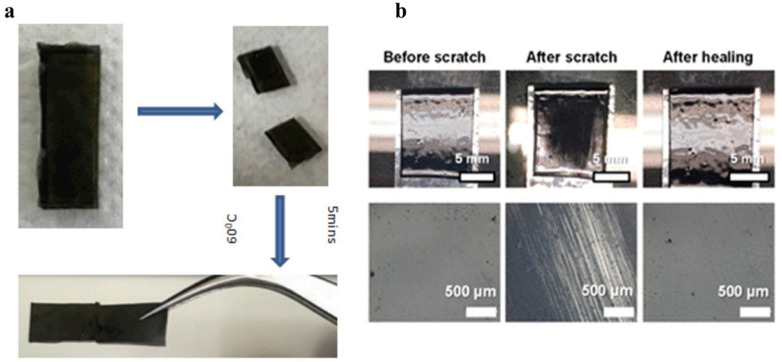
(**a**) Original, cut, and healed graphene oxide vitrimer composite. Adapted with permission from Ref. [85], Copyright 2020, Elsevier. (**b**) Optical images of composites (0.2 wt % CNT) before and after healing @ 90 °C. Adapted from Ref. [86] with permission, Copyright 2020, American Chemical Society.

Unreinforced thermoset polymeric composites used in engineering applications are prone to damage in service due to the inferior mechanical strength of the polymer matrix compared to the carbon reinforcement. Damage from cracks, delamination, scratches, and corrosion significantly reduce the mechanical characteristics of composite materials and can also impact electrical properties in specific applications like electronic devices [79]. Vitrimer composites utilize dynamic covalent bonding to enhance thermoset repair capabilities. Lost performance that occurs when the original covalent bonds are broken will be mostly recovered by the restoration of the dynamic covalent bonds. Vitrimers have a notable feature where reversible crosslinking reactions occur at faster rates above a certain temperature, allowing for self-healing ability in that condition. Pressure must be exerted throughout the repair process of vitrimers to ensure proper contact at the interface for the standard healing process [65,80,81,82,83]. Wu et al. [84] developed epoxy vitrimers through transesterification with a crosslinked structure with primary amines and an excess of hydroxyl groups. This modification results in an increased bond exchange rate, enhancing the healing capabilities of the material. The study found that cracks in the matrix and delamination cracks in the composite can be effectively repaired under high temperatures and pressure. Self-repair at a lower temperature may be achieved by creating vitrimers with dynamic covalent bonds that have lower activation energies and lower Tg. Krishnakumar et al. [85] developed a catalyst-free epoxy vitrimer reinforced with graphene oxide (GO) with aromatic disulfide bonds. The incorporation of GO decreased the Tg of the vitrimer, facilitating self-healing at lower temperatures. Optimal healing occurred at 60 °C for 5 min as shown in Figure 10a. Damage on the multiwall CNT/poly (hindered urea) tends to be easily healed at 90 °C without the need for external forces, as depicted in Figure 10b [86].

**Figure 11:**
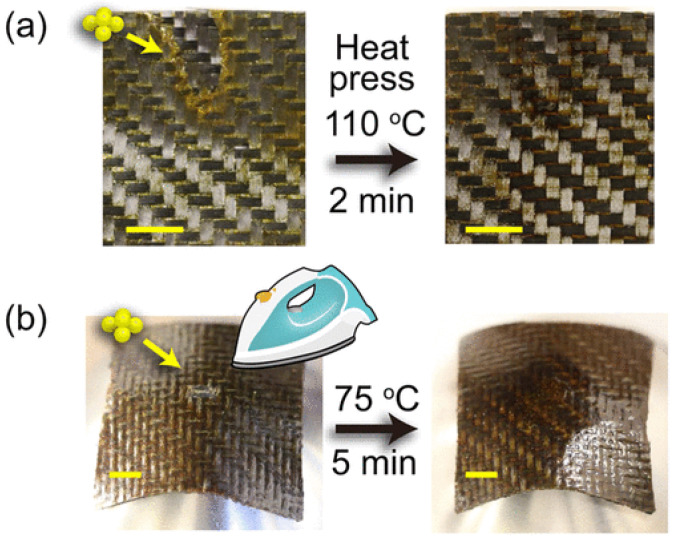
Repairing carbon fiber vitrimer composite with surface damage. (**a**) Rapid repair of flat composite achieved using hot press at 110 °C and 0.01 MPa. (**b**) Mold-free repair of curved composite achieved using handheld iron at ~75 °C. Adapted from Ref. [58] with permission, Copyright 2021, American Chemical Society.

Yu et al. [58] presented a unique healing procedure for carbon fiber-reinforced vitrimer composites that used dynamic imine bonds that reversibly break and reform upon heating. The polyimine vitrimer may be broken down by a mixture of diethylenetriamine (DETA) and ethanol, with subsequent dissolution at room temperature. To repair the damaged surface, polyimine vitrimer powder is applied on its surface and heated to trigger the reaction that is shown in Figure 11. Following this distinctive treatment, the mechanical robustness of the restored composites can be fully regained [58]. It involves repairing damaged materials with the help of raw materials through dynamic imine bonds, rather than relying on pressure and single bond exchange. A comparable method leads to increased bonding strength and reduced pressure compared to direct bonding through hot-pressing [87].

Furthermore, some microcracks may be deeply embedded in a composite and imperceptible to the naked eye due to the unavoidable manifestation of fatigue during the service, resulting in lowered mechanical properties of the composites. Along this line, Koratkar et al. [69] demonstrated the potential of reversing fatigue damage by repeatedly applying heat over Tv to fatigue-damaged carbon fiber/vitrimer composites. Their findings show that failure due to fatigue in the composites was healed regularly with simple heating, significantly increasing the service duration and longevity of the material. However, it should be highlighted that the vitrimer materials utilized in this process must be thermally stable to avoid permanent shifts in the chemical structure due to repeated heating.

### 3.5. Recycling of Vitrimer Composites

**Figure 12:**
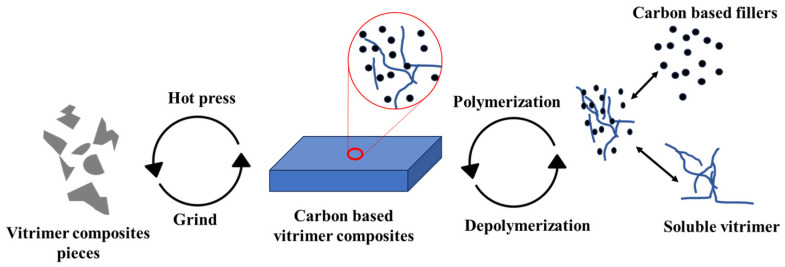
Pictorial representation of recycling carbon-based vitrimer composites.

Owing to dynamic covalent bonding, vitrimers have a relatively high ability to be recycled. This characteristic is particularly useful in carbon-based vitrimer composites. In fact, the environmental requirement and willingness to recycle carbon products are increasing [88]. There are two methods for recycling vitrimer composites: hot-pressing, also known as physical recycling, and cleaving dynamic linkages in solvents, also known as chemical recycling [89]. The hot-pressing technique is quite simple to perform but it can only recycle the entire composite-matrix and reinforcement. The second method allows for separate recycling of the vitrimer matrix and the reinforced carbon fillers but necessitates relatively complex processes and the use of many chemicals [25,33,72], as shown in Figure 12.

**Figure 13:**
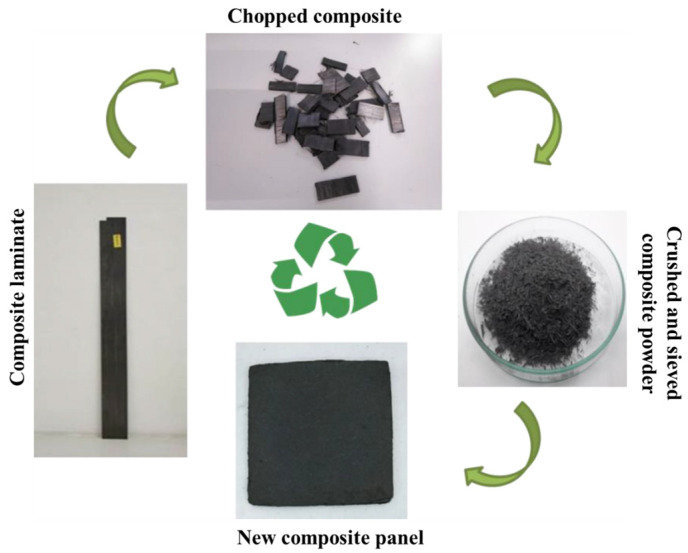
Recycling carbon fiber-based vitrimer composites by the hot-press technique. Adapted with permission from Ref. [90], Copyright 2021, Elsevier.

In the hot-press recycling method, the vitrimer composite specimen is first cut and ground into powder, which is then put in a mold cavity and pressed above the Tv temperature. Comparing the mechanical characteristics of the original and recycled samples can ensure the effectiveness of recycling. If the samples are recycled many times and the chemical structure does not degrade, the hot-pressing method is a highly effective and successful method for recycling vitrimer composite [41]. The hot-pressing technique is not suitable for recycling carbon fiber-reinforced composites since chopping the composite destroys the properties of the carbon fiber. If it is not necessary to maintain the mechanical characteristics of the material with intact reinforcing fibers, then it can be hot-pressed and recycled to produce short fiber-reinforced composites. Rekondo et al. [90] investigated the hot-pressing method for a carbon fiber-based composite with disulfide bonds, produced by pultrusion. As illustrated in Figure 13, the composites were chopped into small pieces, crushed, and re-formed in a mold to produce a composite sheet with recycled short carbon fiber. The tensile strength and tensile modulus were still good, 33.2 MPa and 16.1 GPa, respectively, and usable in diverse industries. However, they were significantly less than those of the original samples (1460 MPa and 144 GPa). Furthermore, when the chemical structure of the matrix is broken due to aging, corrosion, and impurities, but the inner carbon filler remains unaltered, it is extremely desirable to recycle high value carbon fillers separately from the composites.

**Figure 14:**
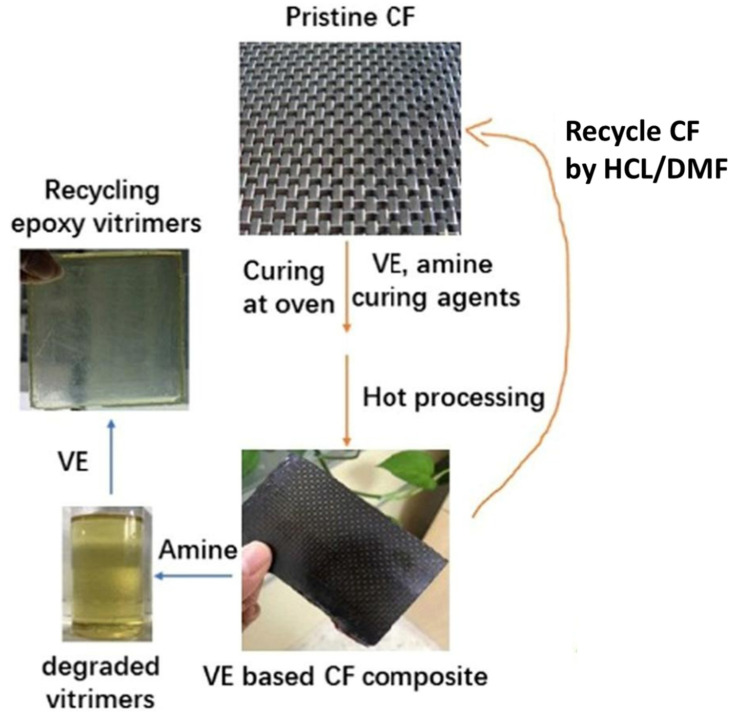
Chemical recycling in the acid solution at room temperature. CF = carbon fiber, VE = vinyl ester, HCL = hydrochloric acid, DMF = dimethylformamide. Adapted with permission from Ref. [94], Copyright 2022, Elsevier.

Recycling vitrimer composites by breaking down the dynamic bonds of the vitrimer in diluents has unique and practical advantage over hot-pressing since the carbon fillers and matrix can be recycled separately. The dissolving approach is ideal for retrieving carbon fibers from vitrimer matrices while retaining their attractive properties (physical, chemical, mechanical) [85,91,92,93]. To accomplish this, a suitable solvent that can rapidly remove the dynamic bonds while not affecting the characteristics of carbon fiber throughout the dissolution method must be carefully selected. For example, ethylene glycol is frequently suggested to break the ester bonds, and it can be used for dissolving polyester- and epoxy-based vitrimers to retrieve carbon reinforcement [50,52,85]; amine solvents like diethylenetriamine and ethanolamine are frequently used to open dynamic imine bonds [39,51]. In the meantime, acidic solutions can damage the Schiff base structure. Wang et al. [94] verified that carbon fiber may be recycled from vanillin-based epoxy vitrimer at ambient temperature in a 0.1 M hydrochloric acid (HCl) solution without losing functionality or chemical structure, as shown in Figure 14. The carbon fiber reinforcement in bio-epoxy vitrimer with dynamic imine linkages is recycled by immersing it in a room-temperature HCl/dimethylformamide solution [40,94,95]. Excluding organic solvents, Zhang et al. [66] developed an epoxy vitrimer that is generated by the interaction of an anhydride and a carefully prepared epoxy prepolymer, which has built-in hydroxy and tertiary amine groups that can degraded quickly in water at temperatures over 160 °C. In composites containing three layers of carbon fiber (43 wt%), the tensile strength reached 356 MPa [66].

**Figure 15:**
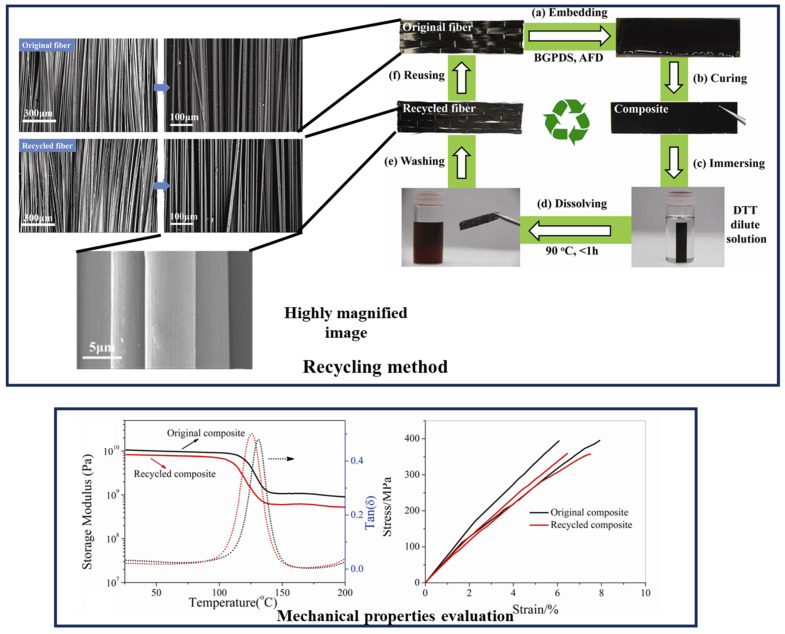
Recycling carbon fiber epoxy vitrimer composites and comparison of fiber quality and strength evaluation of composites (before and after recycling). Adapted with permission from Ref. [63], Copyright 2020, Elsevier.

Furthermore, the decomposed vitrimer network may be repolymerized if the structure of the vitrimer was not deteriorated prior to the recycling process, and once the cleaving agents have been vaporized and removed to form the renewed pure vitrimer [96]. The recycling process, shown in Figure 15, is a carbon-fiber/epoxy system with dynamic ester bonds that are completely recycled for both carbon fillers and vitrimer matrix. The mechanical properties of the regenerated composite were almost same as the original one [63]. The same closed-loop technique has been shown to work for carbon fiber vitrimer composites with dynamic imine bonding [91,97].

## 4. Conclusions and Future Aspects

In summary, this review elucidates the many approaches employed in the preparation, recycling, and reutilization of carbon fiber-based vitrimer composites that have been developed over the last ten years to create thermosets that are sustainable, functional, and exhibit exceptional performance. The production and recycling procedures for vitrimer-based composites reinforced by non-carbon-based fillers (such as glass fibers) and carbon-based fillers share many similarities. Therefore, this review aims to offer crucial and thorough insights into the growth of carbon fiber vitrimer-based composites, while also fostering further advancements within this field. Currently, researchers have conducted investigations into the utilization of high-performance and highly recyclable carbon vitrimer composites in various domains, including electromagnetic shielding [81], sensors and actuators [38], robots [65], and capacitors, in addition to their conventional roles as structural materials and recycling materials. Carbon-reinforced vitrimer composites have significant application requirements in different high-tech domains including electronics, automobile, spacecraft, etc. This is primarily due to their inherent characteristics such as self-healing nature, ability to be reprocessed, and lightweight nature. Undoubtedly, the advancement of vitrimer composites will continue to deepen and expand. 

Notwithstanding the advancements achieved thus far, a substantial distance remains before these materials can be commercially prevalent or supplant the current carbon-reinforced thermosets. In upcoming research, the succeeding two factors are worthy of further consideration. The primary objective of incorporating carbon fillers is to enhance functionality and performance. Simultaneously, fillers have an impact on the dynamic covalent reaction occurring within a vitrimer matrix. As previously mentioned, the incorporation of carbon-based fillers can either facilitate or impede network rearrangement depending on the circumstances. Nonetheless, the impact of various additives on the process of network rearrangement requires additional investigation. Exploiting and carefully considering the cross-influence of carbon-based fillers on the topological rearrangement of vitrimer composites in the areas of restricting polymer chain movement, enhancing thermal properties surplus functional groups on the reinforcement, or other unidentified features is worthwhile. Ultimately, such efforts would ascertain whether mechanical reinforcement and ability to be reprocessed are incompatible.

Furthermore, the initiation of dynamic covalent bonds always has an impact on material properties and thermal stability. Additionally, the prevalence of high-temperature creep resistance is a significant determinant that impacts the practical implementation of vitrimer composites as substitutes for thermoset materials. The detrimental consequences of elevated temperatures potentially include a significant reduction in mechanical characteristics and the annihilation of the initially formed shape, which can lead to critical structural failure issues. This is particularly true for carbon-reinforced vitrimer composites, which are predominantly employed as structural materials. How to maximize creep resistance while maintaining excellent processability is, therefore, a central question in the entire vitrimer field. Approaches to resolving this problem include the development of switchable dynamic bonds, the pursuit of strong dynamic covalent bonds that have substantial activation energies, and the application of light-triggered associative networks, among others [98,99]. Significantly, the fields of dynamic covalent chemistry, vitrimers, and carbon filler materials continue to experience accelerated development. The development and innovation of carbon fiber vitrimer composites are certain to be revitalized by the rise and collision of novel substances and characteristics. Simultaneously, the advancement of manufacturing technology—an endeavor forged in collaboration among engineers and scientists from diverse fields such as chemistry, physics, and material science—will facilitate the widespread adoption of these potentially environmentally friendly, high-performing, and functional polymer composites.
